# Tomographic Imaging of a Forested Area By Airborne Multi-Baseline P-Band SAR

**DOI:** 10.3390/s8095884

**Published:** 2008-09-24

**Authors:** Othmar Frey, Felix Morsdorf, Erich Meier

**Affiliations:** Remote Sensing Laboratories, University of Zurich, Winterthurerstrasse 190, CH-8057 Zurich, Switzerland E-mails: othmar.frey@geo.uzh.ch, felix.morsdorf@geo.uzh.ch, erich.meier@geo.uzh.ch

**Keywords:** Tomography, multi-baseline interferometry, time-domain back-projection, synthetic aperture radar (SAR), airborne SAR, P-band, E-SAR

## Abstract

In recent years, various attempts have been undertaken to obtain information about the structure of forested areas from multi-baseline synthetic aperture radar data. Tomographic processing of such data has been demonstrated for airborne L-band data but the quality of the focused tomographic images is limited by several factors. In particular, the common Fourier-based focusing methods are susceptible to irregular and sparse sampling, two problems, that are unavoidable in case of multi-pass, multi-baseline SAR data acquired by an airborne system. In this paper, a tomographic focusing method based on the time-domain back-projection algorithm is proposed, which maintains the geometric relationship between the original sensor positions and the imaged target and is therefore able to cope with irregular sampling without introducing any approximations with respect to the geometry. The tomographic focusing quality is assessed by analysing the impulse response of simulated point targets and an in-scene corner reflector. And, in particular, several tomographic slices of a volume representing a forested area are given. The respective P-band tomographic data set consisting of eleven flight tracks has been acquired by the airborne E-SAR sensor of the German Aerospace Center (DLR).

## 1. Introduction

In a conventional synthetic aperture radar (SAR) image multiple back-scattering elements distributed along the elevation component are projected to the two-dimensional slant-range plane. With Pol-InSAR techniques only a very limited number of different scattering elements can be localized within a resolution cell. Tomographic processing of SAR data, however, allows resolving the ambiguity in the elevation component and is therefore suitable to produce true three-dimensional images. Hence, different back-scattering elements within a volume can directly be localized. This property can be exploited for the reconstruction of volumetric structures, such as forested areas, as well as for a more detailed imaging of built-up areas and mountainous regions, which exhibit a high percentage of layover regions.

Tomographic processing of SAR data requires that the synthetic aperture in azimuth be extended by a second dimension in direction orthogonal to the plane spanned by the vectors in azimuth and the line of sight. The sampling in this direction, called the normal direction, is realized by coherently combining the data of a sufficient number of adequately separated flight tracks. The common Fourier-based SAR processing algorithm, the SPECAN (SPECtral ANalysis) approach, which has been used in [1], requires that the synthetic aperture be sampled regularly and densely. In reality, the sampling spacing is not uniform in case of airborne SAR data of multiple acquisition paths, and the synthetic aperture in the normal direction is sampled sparsely. As a result the tomographic image is subject to defocusing, high side lobes and ambiguities in the normal direction. In order to overcome the ambiguity problem and to improve the resolution modern spectral estimation methods have been proposed as a substitute to spectral estimation by FFT. These methods include spectral estimation by the Capon method [2] and subspace-based spectral estimators such as the MUSIC algorithm [3][4]. These methods replace the last step, the spectral estimation by FFT, but any geometric approximation made in a previous processing step is still present in the data. We adopt a time-domain back-projection (TDBP) processing technique, which maintains the entire three-dimensional geometric relationship between the measured sensor positions and the illuminated area while focusing the data. So, the key feature of the TDBP approach is an accurate handling of the complex geometry of multi-baseline airborne SAR data. An extensive airborne SAR campaign has been accomplished in September 2006. Two fully polarimetric tomographic data sets - an L-band and a P-band data set - of a partially forested area have been acquired by the German Aerospace Center's E-SAR.

In the next section, the Fourier-based SPECAN approach is revised in order to highlight the approximations that are involved. The same framework is also used to derive the sampling constraints and the spatial resolution for the design of the tomographic experiment and the data processing in the normal direction. Then, the formulation of the TDBP algorithm for tomographic processing is given. Further, the measurement set-up of the tomographic P-band SAR experiment is described and the focusing quality is assessed by analysing simulated and real point targets. Finally, the results obtained from the TDBP-based tomographic reconstruction of a forested area from the E-SAR P-band data are presented.

## 2. The SPECAN Algorithm, Resolution and Sampling in the Normal Direction

For the first demonstration of airborne L-band SAR tomography [1] the three-dimensional focusing of the data was accomplished by a combination of the extended chirp scaling algorithm [5], which was used to focus each data track in range and azimuth direction, and the SPECAN algorithm, which was applied to focus the data in the normal direction. The SPECAN approach was originally designed for azimuth compression of ScanSAR data. The peculiarity of this algorithm lies in the fact that the focused data is obtained by a Fourier transform after a deramping operation.

We want to look again in some detail at the derivation of the SPECAN algorithm for focusing in the normal direction for two reasons: first, to highlight the approximations that are involved in the SPECAN approach, and second, because it provides a good framework to derive two important parameters, the *spatial resolution δ_n_* and the *Nyquist sampling spacing d_n_* in the normal direction.

The model that is used to derive these parameters follows to a large extent the derivation presented in [1]. However, the signal model is loosely based on the derivation of the SPECAN algorithm for azimuth focusing as it is presented in [6].

**Figure 1.**
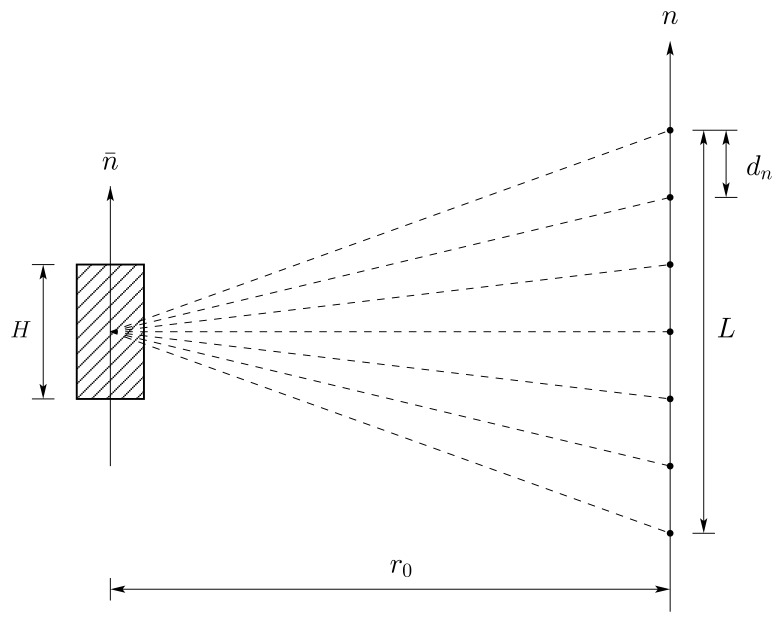
Simplified tomographic imaging geometry after [1]. A volume is illuminated from different positions along a synthetic aperture in normal direction *n*. Each position in normal direction corresponds to a sensor path in azimuth direction. The sensor paths are separated by a constant sampling spacing *d_n_*. The maximal height of the volume is *H. L* is the length of the synthetic aperture in normal direction.

The simplified tomographic acquisition geometry that forms the basis for the derivation of the spatial resolution and the sampling constraints in normal direction *n* – i.e. orthogonal to the plane spanned by the slant-range direction and the azimuth direction – is depicted in Fig. 1. *r_0_* is the range distance at the point of closest approach along the synthetic aperture in normal direction *n*. Equally spaced baselines *d_n_* are assumed and the variation of the off-nadir angle is neglected, so, the vector *n⃗* in normal direction is assumed to be invariant for all acquisition paths. Target coordinates are identified by a bar above the symbol. Assuming that the synthetic aperture in the normal direction *n* is continuous - imagine an infinite number of single look complex images, represented by *s_r_*, acquired from an infinite number of different, parallel flight tracks along *n* - the focused signal in normal direction *υ* (*n̄*_0_) at position *n̄*_0_ in the object space can be written as the following convolution in the time domain:

(1) $$\upsilon \;({\bar{n}}_{0}\;)=\int_{-L/2}^{L/2}s_{r}\;({\bar{n}}_{0}-n\;)h\;(n\;)dn$$

This is equivalent to:

(2) $$\upsilon \;({\bar{n}}_{0}\;)=\int_{{\bar{n}}_{0}-L/2}^{{\bar{n}}_{0}+L/2}s_{r}\;(n\;)h\;({\bar{n}}_{0}-n\;)dn$$

*L* is the length of the synthetic aperture in normal direction *n. s_r_* is the demodulated, received signal. *h* is the matched filter, i.e. the time-reversed reference function, which can be written as:

(3) $$h\;({\bar{n}}_{0}-n\;)=\exp\left(\frac{ik}{r_{0}}{\;({\bar{n}}_{0}-n\;)}^{2}\right)$$

This formulation implements a quadratic phase history, which is obtained by approximating the hyperbolic range history by a second order Taylor series expansion about the point *n* = *n̄*_0_:

(4) $$r\;(n,{\bar{n}}_{0}\;)=2\sqrt{r_{0}^{2}+{\;({\bar{n}}_{0}-n\;)}^{2}}≃2r_{0}+\frac{{\;({\bar{n}}_{0}-n\;)}^{2}}{r_{0}}.$$

*r*(*n*, *n̄*_0_) is the two-way path length between the sensor at position *n* and a back-scatterer within the observed volume at height *n̄*_0_, with a range distance *r*_0_ at the point of closest approach. Inserting eq. (3) into eq. (2) and expanding the quadratic phase term yields:

(5) $$\begin{array}{l} \upsilon \;({\bar{n}}_{0}\;)=\exp\left(\frac{ik}{r_{0}}{\bar{n}}_{0}^{2}\right)\cdot \\ \int_{{\bar{n}}_{0}-L/2}^{{\bar{n}}_{0}+L/2}\underset{s_{d}\;(n\;)}{\underset{︸}{s_{r}\;(n\;)\exp\left(\frac{ik}{r_{0}}n^{2}\right)}}\exp\left(-\frac{i2k}{r_{0}}{\bar{n}}_{0}n\right)dn. \end{array}$$

The exponential within the underbraced term in eq. (5) can be interpreted as a deramping operation which leads to the deramped signal *s_d_*. Then, the whole integral is equivalent to a Fourier transform of the deramped signal *s_d_*:

(6) $$\begin{array}{l} \upsilon \;({\bar{n}}_{0}\;)=\exp\left(\frac{ik}{r_{0}}{\bar{n}}_{0}^{2}\right)\;\int_{{\bar{n}}_{0}-L/2}^{{\bar{n}}_{0}+L/2}s_{d}\;(n\;)\exp\left(-\frac{i2k}{r_{0}}{\bar{n}}_{0}n\right)dn. \end{array}$$

So, in practice, the focused image *υ*(*n̄*_0_) can be obtained by applying a FFT to the deramped signal *sd*.

The phase term in the exponent of eq. (6) can be written as:

(7) $$-\frac{2k}{r_{0}}{\bar{n}}_{0}n=-K_{nr}{\bar{n}}_{0}n$$

where $K_{n_{r}}=\frac{2k}{r_{0}}$ is interpreted as the spatial frequency modulation rate of the signal in normal direction. As it is well known from pulse compression of linear FM signals in range direction, the resolution in the time domain after compression is given by the reciprocal of the processed bandwidth, which is the product of the FM rate and the integration time. Translated to the normal direction and expressed in the spatial domain, the spatial resolution *δ_n_* is the inverse of the product of the spatial frequency modulation rate *K_nr_* and the integration path *L* times 2*π*:

(8) $$\delta_{n}=\frac{2\pi }{K_{nr}\cdot L}=\frac{2\pi }{\frac{2.2\pi }{r_{0}\lambda }\cdot L}=\frac{\lambda r_{0}}{2L}.$$

The Nyquist sampling spacing *d_n_* in normal direction is equivalent to the inverse of the spatial bandwidth *k_n_* times 2*π*, where $K_{n}\;({\bar{n}}_{0}\;)K_{nr}\cdot {\bar{n}}_{0}=\frac{2k}{r_{0}}{\bar{n}}_{0}:$

(9) $$d_{n}\;({\bar{n}}_{0}\;)\leq \left|\frac{2\pi }{k_{n}\;({\bar{n}}_{0}\;)}\right|=\frac{2\pi }{k_{nr}\cdot {\bar{n}}_{0}}=\frac{\lambda r_{0}}{2{\bar{n}}_{0}}.$$

Eq. (9) describes the relationship between sampling spacing and the maximal height ${\bar{n}}_{0}$ = *H* of the imaged volume that can be reconstructed unambiguously:

(10) $$d_{n}\;({\bar{n}}_{0}=H\;)\leq \frac{\lambda r_{0}}{2H}.$$

The respective values for the nominal resolution *δ_n_* and the unambiguous height *H*, which correspond to the tomographic P-band data set presented in this paper, are given in Table 2.

## 3. 3D Focusing in the Time-Domain

In [7] an algorithm has been proposed which is based on single look complex images processed by the extended chirp scaling algorithm including aircraft motion compensation to a straight line. However, instead of focusing the data by deramping and spectral estimation, which would previously involve generating synthetic tracks and a regularization of the samples in the normal direction, a time-domain beamformer (TDB) was applied to focus the data in the third dimension. Every voxel within the volume is focused by a so-called *ad hoc* reference function as it is also known from time-domain back-projection processing. The focusing quality of the TDB approach was found to be superior to the SPECAN based algorithm presented in [1] for unevenly spaced baselines. But in spite of the fact that the TDB directly accounts for the irregular track distribution in normal direction it is still based on artificial, linearized flight tracks, which lie in parallel to each other and which do not represent the true geometry of the flight tracks. In [8] an enhanced method for tomographic focusing of multi-baseline airborne SAR data has been proposed. The core improvement consists of an approximative height-dependent motion compensation and coregistration (HMCC) method. The HMCC approach starts with a stack of range- and azimuth-focused SAR images, which were processed using the extended chirp scaling algorithm including motion compensation with respect to a fixed reference terrain height. Thus, the height dependent motion compensation and coregistration approach is applied to the already focused 2D images. The HMCC approach consists of a so-called *un-moco* step where the motion compensation to the nominal (linearized) track is undone. Then for each 2D SAR image and each height occurring in the tomogram a new post-processing motion compensation (*re-moco*) to the nominal track is carried out with respect to the height under consideration. Eventually, the images are coregistered according to the reference height, to which they have been post-processed. Having applied this HMCC method to an L-band multi-baseline data set the authors of [8] report a considerable improvement in focusing quality compared to the tomographic processing without any height dependent corrections.

We aim at a complete processing in the time domain – after range compression – and focus the data by using the true geometry of the irregularly sampled tomographic acquisition pattern. I.e., every voxel of the 3D SAR image is focused based on the true acquisition geometry, limited only by the accuracy of the navigation data and uncompensated propagation delays. A TDBP processor, which has been tested with airborne [9] and spaceborne SAR data [10], has been extended in order to work with a two-dimensional synthetic aperture. The key idea is that the geometric relationship between every sensor position and the illuminated volume is maintained during focusing without introducing any geometric approximations. Following the signal model presented in [10] the back-projected signal *s_k_* corresponding to the flight track *k* can be expressed as a function of the position *r⃗_i_* on the reconstruction grid:

(11) $$s_{k}\;({\vec{r}}_{i}\;)=\sum_{j=a_{k}\;({\vec{r}}_{i}\;)}^{b_{k}\;({\vec{r}}_{i}\;)}g_{k}\;(R_{k_{ij}},{\vec{r}}_{S_{jk}}\;)\cdot R_{k_{ij}}\cdot \exp\;(i2k_{c}R_{k_{ij}}\;).$$

*r⃗_i_*: position vector of the target*a_k_*, *b_k_*: indices of first, last azimuth position of the sensor within the synthetic aperture of the target position *r⃗_i_*
$\vec{r}S_{jk}$: position vector of the sensor, *j* ∈ [*a_k_*, *b_k_*]*R_kij_*= |
$\vec{r}i-\vec{r}S_{jk|}$ : range distance*gk*(.): range-compressed signal of data track *k**k_c_*= 2 *π fc*/ *c* : central wavenumber*f_c_*: carrier frequency*c*: speed of lightBy extending the coherent addition of the signal contributions to the normal direction the back-projected signal *υ* is obtained, which maps the volume at the position *r⃗_i_*:

(12) $$\upsilon \;({\vec{r}}_{i}\;)=\sum_{k=1}^{m}\sum_{j=a_{k}\;({\vec{r}}_{i}\;)}^{b_{k}\;({\vec{r}}_{i}\;)}g_{k}\;(R_{k_{ij}},{\vec{r}}_{S_{jk}}\;)\cdot R_{k_{ij}}\cdot \exp\;(i2k_{c}R_{k_{ij}}\;),$$

where *m* is the number of flight tracks that build the tomographic pattern. The boundaries of the synthetic aperture in azimuth direction, *a_k_* and *b_k_*, vary as a function of the grid position *r⃗_i_*. This means that we sum up the contributions from those sensor positions $\vec{r}S_{jk}$ which actually build the synthetic aperture for the grid position *r⃗_i_*. Note that an appropriate interpolation procedure (interpolation by FFT) is required in order to retrieve the data values at the correct range distances because of the discrete representation of the range-compressed data.

## 4. Experimental Set-Up

An extensive airborne SAR campaign has been carried out in September 2006. Two fully polarimetric tomographic data sets - a P-band and an L-band data set - of a partially forested area have been acquired by the German Aerospace Center's E-SAR system. Eight corner reflectors were deployed for geometric and radiometric calibration purposes. The positions of the corner reflectors were measured by carrier-phase differential GPS. In the following, we restrict ourselves to describing the results obtained so far with the P-band data set.

The relationship of the sampling spacing *d_n_* in normal direction and the height *H* of the volume that may be imaged unambiguously is given by eq. (10), and spatial resolution *δ_n_* is given by eq. (8). Given a limited number of sensor passes a trade-off has to be made between the achievable resolution and the maximal unambiguous height *H*.

**Figure 2.**
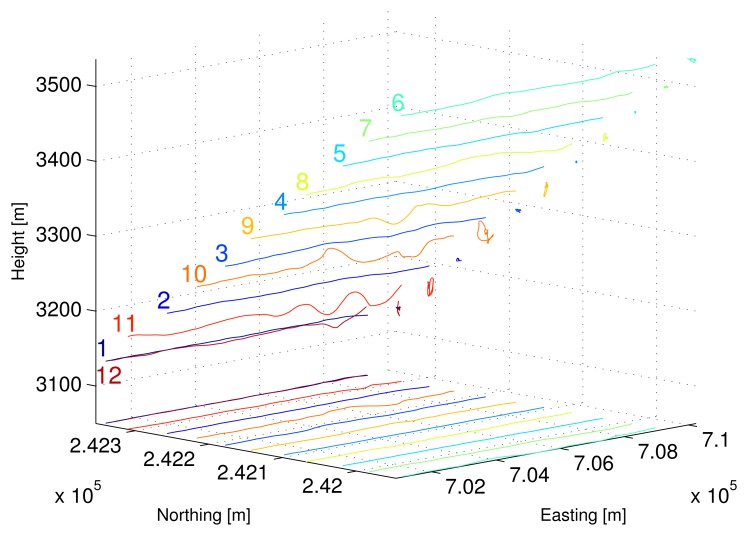
P-band tomographic acquisition pattern consisting of 11 flight tracks + 1 control track. The flight direction is from east to west and the sensor is left-looking. In addition to the actual flight tracks, their projections to the horizontal plane and to the northing-height plane are depicted.

For the design of our experiment the total number of flight tracks that span the synthetic aperture in normal direction was limited to 11 tracks. We have chosen a regular distribution of the flight tracks orthogonal to a mean line of sight at an off-nadir angle of 45° (see Fig. 2) with horizontal and vertical baselines of 40 *m*. This results in a sampling spacing *d_n_* = 56.7 *m* and a synthetic aperture *L* = 567 *m* in the normal direction. Using these figures, a mean range distance *r*_0_ = 3900 *m*, and the wavelength of the carrier signal *λ* = 0.856 *m* we obtain a nominal spatial resolution *δ_n_* ≈ 3 *m* and a nominal unambiguous height *H* ≈ 30 *m* in normal direction.

In Table 1 the parameters of the E-SAR P-band system are summarized. Note that the reduced chirp bandwidth of only 70 MHz in the P-band is due to restrictions imposed by the Swiss Federal Office of Communications to prevent interference of the radar signal with existing RF communication services within the band 390-395 MHz. The nominal chirp bandwidth would be 94 MHz. The E-SAR system is equipped with a modern computer-controlled CCNS4 navigation system combined with a highly precise DGPS/IMU system of the type AEROcontrol IId, both by IGI mbH. The relative positioning accuracy is approximately 0.01 m RMS (see [11]), the accuracies of the attitude angles are given as *σ_θr_* = *σ_θp_* = 0.004° RMS for roll and pitch angle and *σ_θh_* = 0.01° RMS for the heading, and the velocity is measured with an accuracy of *σ_V_* = 0.005 m/s [12].

The TDBP-based tomographic processing of the P-band data set as presented in this paper does not include a point target based phase calibration. For the E-SAR P-band system the carrier frequency is *f_c_* = 350 MHz and the respective wavelength is *λ_c_* ≈ 0.86m m. A range error *r_e_* = 0.01 m corresponds approximately to *λ_c_*/80 or equivalently, to a phase error *φ_e_* = *π*/20. Thus, the impact on the tomographic focusing quality is relatively small compared to the L-band case, where the wavelength is shorter by a factor of 3.7 and tomographic processing of the multi-baseline data set is not feasible without appropriate phase calibration.

The 12 P-band data sets were acquired within one air mission. The maximal time span between the first and the last track is approximately 2 h. The tracks of the tomographic pattern were flown in an interleaved manner in case that an unexpected incidence would have caused an untimely abortion of the data acquisition. In Fig. 2 the geometric configuration of the flight tracks for the P-band tomographic data set is shown. The flight direction is from east to west and the sensor is left-looking. In addition to the actual flight tracks, their projections to the horizontal plane and to the northing-height plane are also depicted. The mission was completed by a control track which has the same nominal flight geometry as the first track. This allows assessing the amount of temporal decorrelation between the first and the last track. Table 2 contains a summary of the parameters which characterize the tomographic data set.

As an external reference, a digital elevation model (DEM) derived from airborne laser scanning (Falcon II, Toposys GmbH) is available for comparison of the ground level and a digital surface model (DSM) acquired by the same sensor is also at hand. The airborne laser scanning data were acquired in spring of 2003. It has to be assumed that the deciduous trees were mostly transparent to the laser signal and therefore do not appear in the LiDAR-derived DSM. In view of this limitation a region that is dominated by coniferous trees has been chosen as test area.

## 5. Simulated Data

In order to quantify the performance of the tomographic processing by TDBP two complete tomographic P-band raw data sets have been simulated and then focused. The raw data simulator emulates the true 3D acquisition geometry using the navigation data of the actual flight tracks and the 3D position of a point target. The same system parameters have been used as they are given for the real-world P-band data set acquired by the E-SAR system.

**Figure 3.**
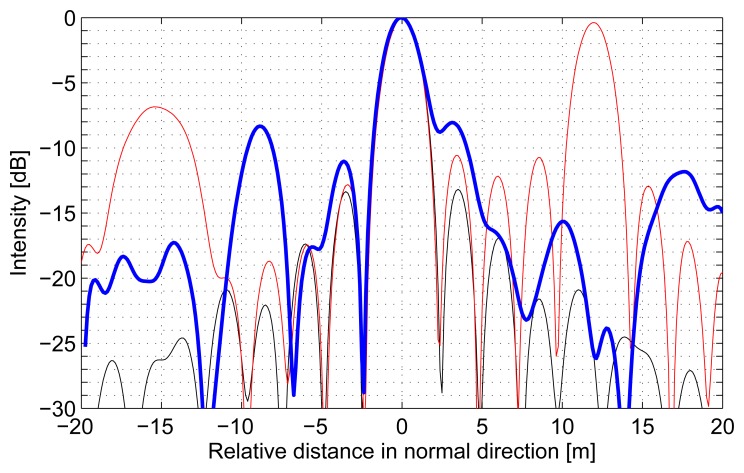
Impulse responses resulting from TDBP tomographic imaging of the multi-baseline P-band raw data set of: a single simulated point target (black line), two simulated point targets which are separated by a distance of 12 m in normal direction (red line), a real, in-scene corner reflector (blue line).

In Fig. 3 the respective impulse responses resulting from TDBP tomographic imaging in the normal direction are given for the three cases: a single simulated point target (black line), two simulated point targets which are separated by a distance of 12 m in normal direction (red line), and a real, in-scene corner reflector (blue line). The simulated data is based on exactly the same geometry as in the real situation. In all cases the point targets are focused properly in terms of resolution (*δ_n_* ≈ 3 *m*). But, the focused signal of the two simulated point targets aligned along the normal direction exhibits a rather strong ambiguous target detection and the focused signal of the in-scene corner reflector shows a considerable amount of anomalous side lobes.

## 6. Experimental Results

**Figure 5.**
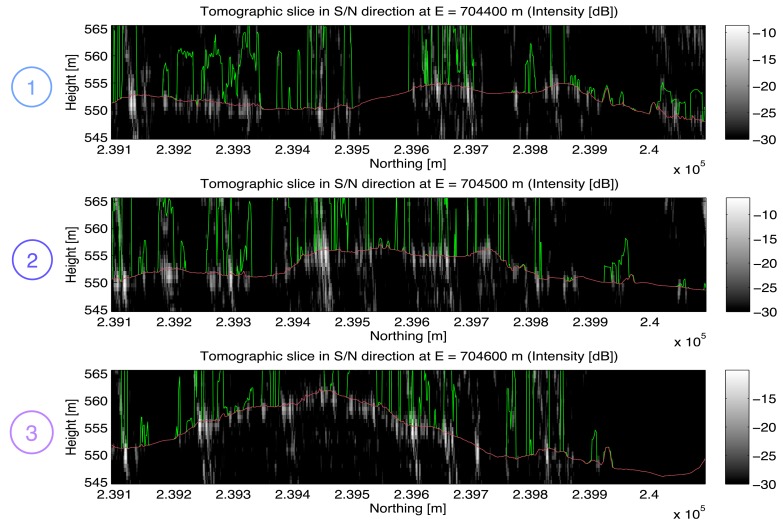
Vertical slices of the SAR tomographic image in *south-northern* direction. Red/green lines in the tomographic slices: High resolution DEM/DSM from airborne laser scanning (Falcon II, TopoSys GmbH) indicating the ground level and canopy height as a reference.

A partially forested area of 400 m × 1000 m size has been selected in order to demonstrate tomographic processing by TDBP using the HH channel of the multi-baseline P-band SAR data set. Since the selected area is relatively flat, a 3D reconstruction grid consisting of a set of *horizontal* layers has been chosen. The voxel spacing is 1 m for both, easting and northing direction, and 1.5 m in vertical direction. A top view of the test area is given in Fig. 5(a) in the form of an orthorectified RGB image. As an external reference data set the LiDAR-based DEM and DSM are used. The RGB ortho-image has been taken at the time of acquisition of the airborne laser scanning data. In Fig. 5(b), a horizontal layer (height level H = 551 m) of the SAR tomographic image is depicted. The coherence map (see Fig. 5(c)) of the zero baseline configuration obtained from track No. 1 and control track No. 12. indicates a low temporal decorrelation for the forested regions of the test area during the total time of data acquisition. All data sets have been processed to zero Doppler centroid frequency.

**Figure 6.**
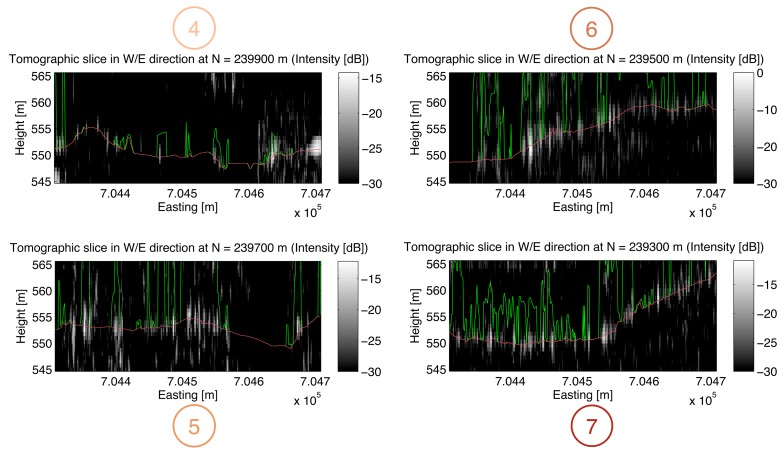
Vertical slices of the SAR tomographic image in west-eastern direction. Red/green lines in the tomographic slices: High resolution DEM/DSM from airborne laser scanning (Falcon II, TopoSys GmbH) indicating the ground level and canopy height as a reference.

Seven vertical tomographic slices of the imaged volume separated by a regular spacing have been selected for visualization: In Fig. 5 three slices (No. 1-3) running in south-northern direction at easting values *E* = 704400 *m*, *E* = 704500 *m*, and *E* = 704600 *m* are depicted and in Fig. 6 four slices (No. 4-7) running in west-eastern direction at northing values from *N* = 239300 *m* to *N* = 239900 *m* are given. For smoother visualization the data have been upsampled in the vertical direction by a factor of 2 after focusing. The tomographic slices represent the measured radar intensity values in dB. The red and the green lines indicate the reference height information obtained from the laser DEM or DSM, respectively.

## 7. Discussion and Conclusion

To the authors' knowledge, for the first time P-band tomographic SAR images of a larger forested area (400 m × 1000 m) have been presented. Compared to previous work in the field of SAR tomography (e.g. [1, 7]) a different 3D focusing concept, namely, a complete time-domain back-projection processing to 3D reconstruction grid, has been successfully applied.

A comparison of the tomographic slices resulting from the airborne multi-baseline P-band data set with the DEM/DSM obtained from laser scanning indicates that high intensity values are predominantly located at the ground level within forested areas. This outcome conforms with what can be expected from horizontally polarized P-band radar back-scattering of a forested area, where double-bounce scattering from the ground surface and tree trunks is a dominant scattering mechanism. Furthermore, this result supports the claim that DEMs obtained by means of P-Band interferometry provide a good estimation of the ground topography underneath canopy.

However, high intensity values within the tomographic images are often accompanied by considerable side lobes and ambiguities in the normal direction.

The simulations show that the point targets are well-focused by tomographic processing using the TDBP algorithm in terms of resolution and separability. The anomalous side lobes that appear for the in-scene corner reflector might be due to a remaining geometric calibration error of the multi-baseline data. If multiple targets are distributed along the normal direction the focused signal is disturbed by ambiguities as a result of the sparsely sampled synthetic aperture in normal direction. This system inherent problem is given by the limited unambiguous height in the normal direction. Therefore, suppression of ambiguities is a main concern in order to further improve the quality of the tomographic images in a future step.

## Figures and Tables

**Figure 4.**
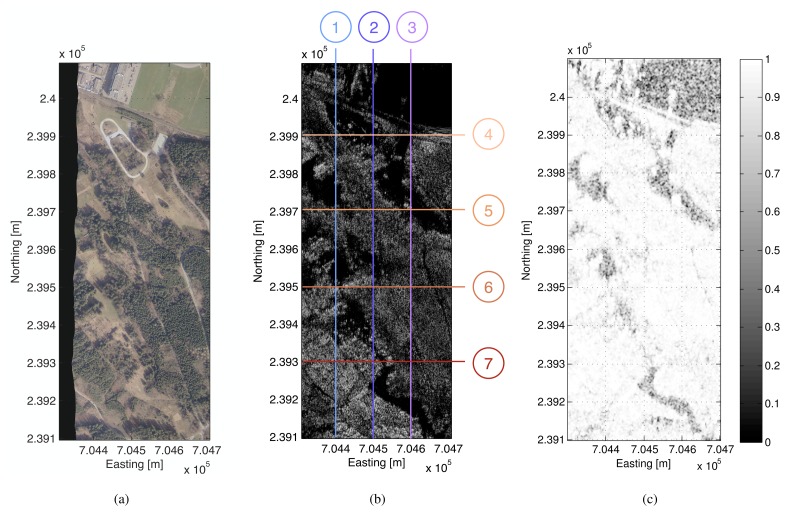
(a) RGB ortho-image of the partially forested area selected for tomographic imaging taken at the time of acquisition of the reference DEM/DSM data sets by airborne laser scanning (Falcon II, TopoSys GmbH), (b) SAR intensity values within a horizontal tomographic slice of the reconstruction grid on the height level H = 551 m. (c) Coherence map of the zero baseline configuration (track no. 1 and track no. 12).

**Table 1.** E-SAR P-band system parameters.

**Carrier frequency**	350 MHz
**Chirp bandwidth**	70 MHz
**Sampling rate**	100 MHz
**Polarizations**	HH-HV-VV-VH
**PRF**	500 Hz
**Ground speed**	90 m/s

**Table 2.** Nominal parameters used in the set-up of the P-band tomographic SAR experiment.

**Number of flight tracks**	11+1
**Nominal track spacing** *d_n_*	56.7 m
**Horizontal baselines**	40 m
**Vertical baselines**	40 m
**Synthetic aperture in normal direction** *L*	567 m
**Nominal resolution in normal direction *δ****_n_*	3m
**Approx. unambiguous height** *H*	30 m
